# The goalpost moves to preserve the gate: productivity, extraction, and indigenous educational values in STEMM

**DOI:** 10.3389/fsoc.2026.1934211

**Published:** 2026-08-12

**Authors:** Karen Colbert

**Affiliations:** Atlas Executive Consulting, Hancock, MI, United States

**Keywords:** gatebreaking, gatekeeping, indigenous education, institutional isomorphism, productivity, relational labor, STEMM equity, tribal colleges and universities

## Abstract

Productivity in science, technology, engineering, mathematics, and medicine (STEMM) is never culturally neutral. Dominant productivity regimes count outputs such as publications, grants, and response times while rendering invisible the relational labor that sustains student persistence, faculty wellbeing, and institutional belonging. This Perspective argues that mainstream productivity norms function as gatekeeping mechanisms sustained by coercive institutional isomorphism, and that relational labor is not service overhead but core scholarly output. Drawing on a 2026 keynote delivered at the American Indian College Fund Tribal College Faculty Convening and recent peer-reviewed literature, I identify five pervasive lies about productivity in STEMM (worth equals output, urgency equals importance, self-sacrifice equals leadership, representation equals transformation, and burnout equals impact) and show how each perpetuates extraction rather than reciprocity. Tribal Colleges and Universities (TCUs) occupy a paradoxical position: pressured to perform mainstream “publish or perish” norms to survive accreditation and funding requirements, yet founded on missions that explicitly center Indigenous revitalization, language reclamation, and cultural sovereignty. The lessons TCUs offer come not from having solved the productivity problem but from their founding documents, which name what mainstream institutions refuse to name: that education exists to sustain people, culture, and community, not to extract from them. When institutions celebrate diverse faculty while refusing to redesign evaluation systems, the goalpost moves to preserve the gate. The article concludes with implications for reimagining faculty evaluation, resourcing invisible labor, and advancing gatebreaking across STEMM ecosystems.

## 1 Introduction: the most dangerous lie in higher education

In February 2026, I stood before an audience of Tribal College faculty at the American Indian College Fund Faculty Convening and asked a question that had been building for years: What if the most dangerous lie in higher education is not that we are unproductive, but that we have accepted someone else's definition of what productivity means? The room recognized the question immediately. These faculty live it daily, caught between institutional demands to publish, secure grants, and demonstrate “impact” through metrics designed for research-intensive universities, and the reality of their work: mentoring students through intergenerational trauma, revitalizing endangered languages, and rebuilding community trust in institutions that once harmed their communities.

The question was not abstract. In 2022 my son was diagnosed with osteosarcoma; we flew across the country to save his leg from amputation. Through that season I kept teaching full time and answering emails from hospital waiting rooms, performing professionalism while my family fought to survive. When the cancer returned in 2025, this time in his arm, I understood something I have never been able to unsee: the institution would continue moving whether my family survived that moment or not. I say that without bitterness. This article is written from that clarity.

This Perspective argues that dominant productivity norms in science, technology, engineering, mathematics, and medicine (STEMM) are not neutral measures of excellence. They encode specific values (individualism over collectivism, speed over depth, extraction over reciprocity) and function as gatekeeping mechanisms that diminish the contributions of any institution or scholar whose work exceeds what the metrics can measure (Hope Hassberg et al., 2022). I advance two related claims. First, the productivity trap is sustained by coercive institutional isomorphism: the pressure organizations face to conform to dominant norms in order to maintain legitimacy and resources (Cardona Mejía et al., 2020; Rusch and Wilbur, 2007). Second, the missions and founding documents of Tribal Colleges and Universities (TCUs) already articulate the alternative that mainstream higher education has spent decades failing to name: that relational labor, including mentorship, community trust-building, and cultural revitalization, is not service overhead but core scholarly output (Brayboy, 2013). When institutions celebrate diverse faculty while refusing to redesign the evaluation systems that judge them, the goalpost moves to preserve the gate. Gatebreaking, the deliberate dismantling of exclusionary structures, begins when institutions stop moving the goalpost and start asking who built it.

This article speaks to two audiences at once. To TCU faculty and administrators who have been told, implicitly or explicitly, that their institutions must adopt the productivity metrics of predominantly white research universities to earn legitimacy: your missions already articulate what mainstream higher education has spent decades failing to name. You do not need to abandon it to be taken seriously. To mainstream institutions that have not yet reckoned with what they are losing by refusing to count the labor that sustains educational mission: the framework you need has existed in TCU founding documents for generations. The question is whether you are willing to learn from it.

A note on positionality: I have taught mathematics within the TCU system for more than 10 years and have pursued formal training in Indigenous educational frameworks through the American Indian College Fund (the College Fund) and the American Indian Higher Education Consortium (AIHEC). The arguments here emerged alongside Indigenous colleagues, students, and community members whose knowledge shapes every claim; they do not speak for Indigenous academic communities as a monolith, and I invite critique and correction from Indigenous scholars and TCU faculty who have lived the tensions this article describes.

## 2 The productivity paradox

Productivity metrics promise objectivity: clean numbers for administrators, benchmarks for tenure committees, standardized criteria for funders. Yet the more rigorously institutions apply them, the more reliably they reproduce inequity, burnout, and the devaluation of the labor that sustains educational mission (Reynell van der Ross et al., 2022). The metrics privilege what is easily counted (publications, citations, grant dollars) while rendering invisible the relational labor that disproportionately falls to Indigenous scholars, faculty of color, and women: mentoring students through personal crises, serving on diversity committees, translating research for community audiences (Azhar and DeLoach McCutcheon, 2021; Opdycke, 2022). The COVID-19 pandemic exposed the deeper assumption: productivity expectations presume a workforce without caregiving responsibilities, and when caregiving burdens merged with unchanged expectations, relational work became simultaneously indispensable and invisible (Hope Hassberg et al., 2022).

The paradox is sharpest for Indigenous scholars and for TCUs, where the measurement infrastructure itself erases the mission. The disparities are stark. Of American Indian and Alaska Native students who enrolled in postsecondary education, 43% did not persist in 2009 compared with 33% of White students, and only 12% of those who persisted earned at least a bachelor's degree by 2010 compared with 37% of White students (Lopez, 2018). His review shows why conventional models cannot explain these numbers: the factors that actually shape Indigenous student success cluster into family support, institutional support, tribal community support, and academic performance, yet the constructs at the heart of those themes resist conventional measurement. Students' desire to give back to their communities emerged as a primary driver of persistence across the qualitative literature while the quantitative literature almost never detected it, because the instruments were never designed to measure it (Lopez, 2018). Dominant persistence narratives compound the problem by framing Indigenous students through deficit lenses, attributing attrition to individual shortcomings rather than to institutional failure to recognize relational commitments such as giving back to one's nation (Lopez and Tachine, 2021). Federal postsecondary data systems compound the problem, systematically misrepresenting Indigenous student outcomes and rendering TCU missions invisible to policymakers and funders (Lopez et al., 2024). You cannot measure what you have designed a system to ignore.

## 3 Five lies higher education teaches about productivity

The productivity trap is sustained by interlocking institutional narratives: lies not in the sense of conscious deception but of claims presented as universal truths while serving particular interests. Each performs a specific gatekeeping function; together, they constitute a coherent system.

### 3.1 Lie #1: your worth is measured by your output

The foundational lie is that a scholar's value can be counted. Publications, citations, and grant dollars become proxies for human worth, reducing complex intellectual and relational labor to a single measurable dimension. The lie operates through coercive isomorphism: accreditation bodies, funding agencies, and ranking systems push standardized metrics onto vastly different institutional types, homogenizing evaluation from research universities to community-serving Tribal Colleges (Cardona Mejía et al., 2020; Rusch and Wilbur, 2007). The scholar who publishes prolifically but mentors poorly is rewarded; the scholar who transforms students' lives but publishes slowly is penalized. The arithmetic of the credit hour makes the lie concrete. A three-credit mathematics course counts as three credit hours whether it enrolls 10 students or fifty, whether grading is automated or every assignment is returned with individual feedback, and whether students arrive prepared or need their instructor to sit with them through every step. At Tribal Colleges, where small classes and one-to-one relationships are the pedagogy, the emotional labor of teaching 10 students can equal or exceed the labor of teaching thirty, yet the workload accounting recognizes neither. Reducing an instructor's value to the credit hours listed on a syllabus erases precisely the labor that determines whether students persist. TCU missions have always named the alternative: sustaining community, language, and cultural identity is not auxiliary to education. It is education.

### 3.2 Lie #2: urgency equals importance

The second lie conflates speed with significance. Email must be answered immediately; constant availability becomes the marker of professionalism. This is normative isomorphism, the internalization of professional norms that define legitimate academic behavior (Cardona Mejía et al., 2020). The urgent crowds out the important: the 3-h mentoring conversation is invisible in annual review, and the ceremony that grounds a faculty member's teaching and sustains their connection to community is filed under personal activity rather than professional practice (Brayboy, 2013). The contradiction sharpens around assessment. Institutions require that classroom and programmatic modifications be justified through data-informed decisions, and accreditors expect to find the evidence in the files. Yet no time is allotted, and none is valued, for the reflection that data-informed decision-making actually requires. Faculty are expected to collect data, interpret it, modify instruction in real time, and document the evidence, all within semesters that schedule zero hours for reflective practice. Urgency does not merely crowd out the important; it makes the very practice institutions claim to require structurally impossible. When institutions treat relational labor as optional and administrative compliance as mandatory, they are not making a scheduling decision. They are making a values statement.

### 3.3 Lie #3: self-sacrifice is leadership

The third lie reframes exploitation as virtue: the most committed scholars work nights and weekends, absorb dysfunction without complaint, and model devotion as though exhaustion were evidence of excellence. Academia has developed its own version of what sociologists describe as conspicuous consumption: conspicuous productivity, the public performance of exhaustion as proof of value. Overbooked calendars, midnight emails, and performative overwhelm become institutional currency. When leaders model self-sacrifice rather than psychosocial safety, boundary-setting reads as weakness and the actual work of team wellbeing goes unresourced (Sjöblom et al., 2022). Indigenous scholars, faculty of color, and women faculty are disproportionately asked to sacrifice research time for mentoring and student support, praised for it, and then not rewarded for it in promotion decisions (Azhar and DeLoach McCutcheon, 2021). The burden extends beyond identity. Faculty who serve underrepresented communities carry this labor whether or not they belong to those communities themselves, because relational labor follows the students it sustains, not the demographics of the instructor. When sacrifice is framed as leadership, it becomes harder to name as extraction.

### 3.4 Lie #4: representation alone creates transformation

The fourth lie promises that hiring diverse faculty will automatically transform institutional culture. Ahmed (2012) exposes how diversity commitments function as non-performatives, speech acts that create the appearance of change while leaving exclusionary structures intact, and how diversity work is routinely allocated to those who already do not fit institutional norms, producing overwork and containment rather than transformation. Hess (2022) extends the analysis through interest convergence: post-2020 diversity initiatives often reflect performative commitments that serve institutional legitimacy and evaporate once dominant-group interests no longer align with equity goals.

This dynamic produces what I call the moving goalpost problem. When underrepresented faculty are hired, the institution celebrates. When those faculty demand structural change (equitable workload distribution, recognition of relational labor, real decision-making power) the institution reframes the standard: diversity is not enough, we need “excellence”; excellence is not enough, we need “fit”; fit is not enough, we need “collegiality.” The goalpost moves to preserve the gate. TCU founding documents have never accepted this logic. Their responsibility to community is written into their charters, and the goalpost cannot move when the mission is written into the charter.

### 3.5 Lie #5: burnout is the cost of impact

The fifth lie normalizes burnout as the inevitable price of meaningful work, converting a structural problem, institutions extracting unsustainable labor, into a personal failing of resilience or self-care. Empirically, burnout is a structural outcome: among academics studied during COVID-19, perceived organizational support was the strongest predictor of engagement and the strongest buffer against burnout risk (Reynell van der Ross et al., 2022). Faculty carrying disproportionate invisible labor face the highest burnout risk and are the most likely to be told that their exhaustion reflects a lack of professionalism (Azhar and DeLoach McCutcheon, 2021). The institution benefits from their labor until they leave. Then it hires someone new.

## 4 Relationality as infrastructure: the missions argument

TCUs occupy a paradoxical position. Founded to serve Indigenous communities, revitalize languages and cultural practices, and exercise educational sovereignty (Brayboy, 2013), they must nonetheless perform productivity on terms set by accreditors and federal funding formulas built for predominantly white, research-intensive institutions: graduation timelines designed for traditional-age residential students, and metrics that cannot see mentoring through intergenerational trauma or rebuilding community trust in education (Lopez, 2018; Rusch and Wilbur, 2007). From a Tribal Critical Race Theory perspective, these metrics are not neutral measures of quality; they are tools of ongoing colonization that discipline Indigenous institutions into conformity with settler colonial values (Brayboy, 2013; Hextrum et al., 2022). No institution should have to choose between its founding mission and its funding.

The lesson TCUs offer mainstream higher education, however, comes not from having resolved this tension but from their founding documents. I call this the missions argument: a claim not about what any particular TCU does on any given day, but about what these institutions committed to at their founding, and what that commitment reveals about the poverty of mainstream productivity norms. Consider Diné College, the first tribally controlled college in the United States, whose mission is to serve the academic and cultural needs of the Diné people. That mission does not promise merely to produce graduates who can compete in the mainstream economy. It promises to serve an entire people, a promise requiring forms of labor that productivity metrics cannot see, much less measure. The mission statement is itself an act of gatebreaking: a public declaration that the metrics of someone else's excellence do not define what the institution exists to do.

To illustrate how relational infrastructure can be operationalized, I offer the Four Directions of Student Engagement framework, developed through classroom observation in Tribal College mathematics courses serving adult reentry learners and currently under review at the Tribal College and University Research Journal. Grounded in Anishinaabe and Ojibwe teachings of the Medicine Wheel, the framework conceptualizes engagement as a network of four interconnected relationships: student to peers, student to instructor, student to content, and student to culture. Drawing on the language of social network analysis, it asks two questions of each relationship: does it exist, and is it reciprocal? A course can offer strong content, responsive instruction, and opportunities for peer interaction, yet students still disengage when those relationships remain one-directional. When engagement is understood as a network of reciprocal relationships rather than a collection of individual behaviors, many challenges commonly framed as student deficits become visible as problems of educational design, and the relational labor faculty invest in building reciprocity becomes visible as core pedagogical work.

This is not a call for mainstream institutions to appropriate Indigenous frameworks or imitate TCU practices. It is a call to read the missions. To sit with what they name and ask: What would it mean if we said this out loud in our own founding documents? What forms of labor does our mission require that our evaluation systems do not recognize? Whose work is rendered invisible by our productivity metrics? What would change if we centered relationality, reciprocity, and community accountability as measures of excellence? TCU missions have already answered these questions for their own contexts. The invitation to mainstream institutions is not to copy the answers. It is to finally start asking the questions.

The implications are epistemological, not merely administrative. If relational labor is scholarly output, then publication counts are inadequate measures of faculty productivity. If education exists to sustain communities, then graduation rates calculated on 4-year timelines are inadequate measures of institutional success. If cultural revitalization is a core institutional function, then accreditation standards that ignore it are not measuring educational quality; they are enforcing cultural conformity.

## 5 From gatekeeping to gatebreaking: implications

The strongest case for standardized metrics (predictability, accountability, comparability) fails on three grounds. The metrics are not culturally neutral; they encode individualism, speed, and extraction. Their benefits accrue primarily to external stakeholders (accreditors, funders, ranking agencies) while their costs are borne by the communities being evaluated. And metrics that systematically ignore relational labor do not ensure accountability; they ensure the appearance of accountability while obscuring the actual work being done. The alternative is not the absence of accountability. It is accountability to mission, to community, and to the values institutions claim to serve.

Reform requires coordinated agents with distinct leverage. Faculty governance bodies can revise promotion and tenure criteria to recognize relational labor; structured mentoring networks distributed across departments show particular promise for redistributing the invisible labor of student support (Markle et al., 2022). Indigenous education coalitions, specifically AIHEC and the American Indian College Fund, have the standing and the knowledge to develop Indigenous-centered evaluation frameworks and accreditation standards grounded in data sovereignty, not because mainstream institutions have invited that work, but because TCUs can set the terms. Federal policymakers can reform funding formulas and data systems, including IPEDS, to capture Indigenous student experiences and value community-based participatory research (Lopez et al., 2024). What is required of mainstream institutions in these partnerships is not leadership. It is the institutional humility to follow.

## 6 Conclusion: toward a relational paradigm of excellence

Is education an extractive enterprise that measures value by counting outputs, or a relational practice that sustains communities, cultures, and the capacity for collective flourishing? TCU missions answered that question at their founding. The missions argument advanced here is not a new discovery. It is the recognition that the framework has existed for generations, written into the institutional DNA of colleges founded to sustain communities rather than extract from them, and that mainstream higher education has simply refused to read it.

The productivity trap cannot be escaped by working smarter or setting better limits. It is built into the evaluation systems, cultural norms, and incentives of STEMM higher education and sustained by the five lies examined here. The scholars who are burning out, leaving academia, or being denied tenure because their labor is not counted are not failing to meet standards. They are being failed by systems that refuse to count the labor that sustains educational mission. Gatebreaking does not open existing gates wider. It asks who built the gates, and whether what they protect is worth preserving. The framework already exists. It is a mission statement waiting to be read.

Productivity is never culturally neutral. The goalpost moves to preserve the gate. Recognizing this is where gatebreaking begins.

## Author's note

The Four Directions of Student Engagement framework is the author's original scholarly contribution, developed from direct classroom observation at a Tribal College and currently under review at the Tribal College and University Research Journal.

## Data Availability

The original contributions presented in the study are included in the article/supplementary material, further inquiries can be directed to the corresponding author.
